# Research on simulation in radiography education: a scoping review protocol

**DOI:** 10.1186/s13643-020-01531-2

**Published:** 2020-11-21

**Authors:** Mona Vestbøstad, Klas Karlgren, Nina Rydland Olsen

**Affiliations:** 1 Faculty of Health and Social Sciences, Department of Health and Functioning, Western Norway University of Applied Sciences, 5063 Bergen, Norway; 2 The Department of Learning, Informatics, Management and Ethics, Karolinska Institutet, 171 77 Stockholm, Sweden; 3 The Department of Research, Education, Development and Innovation, Södersjukhuset, 118 83 Stockholm, Sweden

**Keywords:** Simulation, Radiography, Education

## Abstract

**Background:**

Today, there are fewer opportunities for health care students and staff for skills training through direct patient contact. The World Health Organization therefore recommends learning about patient safety through hands-on experience and simulation. Simulation has the potential to improve skills through training in a controlled environment, and simulation has a positive effect on knowledge and skills, and even patient-related outcomes. Reviews addressing the use of simulation across the different radiography specialties are lacking. Further knowledge on simulation in radiography education is needed to inform curriculum design and future research. The purpose of this scoping review is to explore, map, and summarize the extent, range, and nature of published research on simulation in radiography education.

**Methods:**

We will follow the methodological framework for scoping reviews originally described by Arksey and O’Malley. We will search the MEDLINE, Embase, Epistemonikos, The Cochrane Library, ERIC, Scopus, and sources of grey literature. A comprehensive search strategy for Ovid MEDLINE was developed in collaboration with a research librarian. An example of a full electronic search from the Ovid MEDLINE (1641 articles records, January 9, 2020) is provided and will be used to adapt the search strategy to each database. Two independent review authors will screen all abstracts and titles, and full-text publications during a second stage. Next, they will extract data from each included study using a data extraction form informed by the aim of the study. A narrative account of all studies included will be presented. We will present a simple numerical analysis related to the extent, nature, and distribution of studies, and we will use content analysis to map the different simulation interventions and learning design elements reported. Any type of simulation intervention within all types of radiography specializations will be included. Our search strategy is not limited by language or date of publication.

**Discussion:**

An overview of publications on simulation in radiography education across all radiography specialties will help to inform future research and will be useful for stakeholders within radiography education using simulation, both in the academic and clinical settings.

**Systematic review registration:**

Open Science Framework (OSF). Submitted on October 18, 2020

**Supplementary Information:**

The online version contains supplementary material available at 10.1186/s13643-020-01531-2.

## Background

New technology and methods for diagnosis and treatment require that health personnel keep abreast with new practices [1]. Traditionally, clinical and communication skills were taught at the bedside [2]. However, in the clinical environment, it is challenging to make adequate observations and to perform feedback and to have enough time for reflection and discussion [2]. Also, today, changes in health delivery have resulted in shorter hospital stays and reduced patient availability for learning [3]. Several other factors make clinical teaching challenging, patients being too sick or unwilling to participate in teaching, and increasing efficiency demands leading to a shorter time for patient consultations [2]. The World Health Organization (WHO) recognizes that patient safety knowledge applies to all areas of practice and to all health care professions ([4], p. 22). To facilitate this, the WHO has provided a Patient Safety Curriculum Guide which recommends learning about patient safety through hands-on experience and simulation ([4], p. 84). Simulation is an important pedagogical method widely used by healthcare professions and may involve a range of learning activities [5]. Motola et al. highlight that simulation is a pedagogical method which has the potential to improve skills and skill retention through training in a controlled environment. Results from systematic reviews show that simulation has a positive effect on learning knowledge and skills [6–8], and can potentially improve patient-related outcomes [6, 9–13].

Issenberg and Scalese [3] state that the aim of the simulation is “to imitate real patients, anatomic regions, or clinical tasks or to mirror the real-life situations in which medical services are rendered.” Different types of simulators are used for simulation: part-task trainers, simulated patients, simulated environments, virtual reality and haptic systems, computer-based systems, and integrated simulators (instructor-driven simulators or model-driven simulators) [14]. Simulation is frequently described as high fidelity or low fidelity [15]. A simulation that offers complex and immersive scenarios by providing realistic feedback is described as high fidelity [16]. The use of a static model or task trainer that feels less real to the learner and offers no or low responsiveness is referred to as low-fidelity simulation [17]. To achieve optimal and efficient utilization of resources when designing simulation-based activities, it is recommended to perform a needs assessment, define learning outcomes, design a scenario to provide the context for the simulation including the levels of fidelity, ensure a facilitative approach, conduct pre-briefing and debriefing and feedback/evaluation, make available resources for preparing the participants, and pilot test the simulation scenario before implementation [18].

Simulation is regarded as a highly suitable strategy for learning radiography [11, 19]. The use of simulation in radiography education has been shown to enhance the radiographers’ perceptions of self-efficacy and critical thinking skills in image evaluation and patient assessment ([19], p.93). The professional practice in radiography is characterized by the use of advanced technologies and equipment for diagnostic purposes or for the treatment of medical conditions [20]. Important skills for simulation-based learning are related to positioning, exposure, physics, patient care, and quality assurance ([19], p.52). Students need opportunities to practice in a safe environment to ensure quality in the profession, and simulation offers the possibility for training without putting the patient at risk [3]. Simulation also offers the benefit of repeated learning of outcomes that promote increased cognitive recall and higher confidence with clinical tasks [21–23]. The term radiographer refers to “professional roles in the fields of diagnostic imaging, nuclear medicine, interventional radiology, and radiation therapy” ([24], p.20).

Simulation in radiography has previously been addressed in a literature review which focused on the simulation of conventional diagnostic radiography [11]. Most studies published after this review were studies with small sample sizes, evaluating different aspects of simulation [5, 21, 25–31]. Several of these studies used mixed methods [27]. Examples of topics covered were related to emotional preparedness when encountering open wounds [31] or when being exposed to clinical burns cases [29], confidence levels before and after simulation [30], and perceptions of learning in different high-fidelity computed tomography simulation environments [27]. Other experimental studies compared the use of virtual reality versus traditional placements [26], virtual reality against existing simulation techniques [25], and virtual reality against clinical role-play [21]. Simulation was also compared against traditional therapeutic radiography placements in a randomized controlled trial [5].

According to Lee et al. [27], reviews addressing the use of simulation across the different radiography specialties are lacking. Further knowledge on simulation in radiography education is needed to inform curriculum design and future research. The aim of this proposed scoping review is to explore, map, and summarize the extent, range, and nature of published research on simulation in radiography education. To achieve the aim of this review, we will:

1. Explore the *extent and range of simulation research* conducted in radiography education (e.g., publication dates, volumes, yearly distributions, proportions, geographical locations)
2. Explore *research methods and designs* used in research on radiography education (e.g., purposes, contexts, study populations, sample sizes, designs, and methods for data collection)
3. Explore *simulation interventions reported* in research on simulation in radiography education

## Methods

We will follow the methodological framework for scoping reviews originally described by Arksey and O’Malley [32] and later advanced by Levac et al. [33] and by Khalil et al. [34]. This framework consists of the following five stages: (1) identifying the research question by clarifying and linking the purpose and research question, (2) identifying the relevant studies using a three-step literature search in order to balance the breadth and comprehensiveness, (3) careful selection of the studies using a team approach, (4) charting the data in a tabular and narrative format, and (5) collating the results to identify the implications of the study findings for policy, practice, or research [34].

We drafted the protocol using the Preferred Reporting Items for Systematic Reviews and Meta-analysis Protocols (PRISMA-P checklist, Additional file 1) [35]. For the scoping review, we plan to follow the newly developed reporting guidelines for scoping reviews: the PRISMA Extension for Scoping Reviews (PRISMA-ScR) [36]. We have registered our protocol in the Open Science Framework. Due to the iterative nature of the scoping review, methodology changes to the protocol can occur. We will report any changes to the protocol.

### Eligibility and exclusion criteria

We will include research publications that involve radiography students, faculty in radiography education and/or clinicians, and publications that describe and/or evaluate any type of simulation intervention within any type of professional radiography specialization. All empirical and theoretical/conceptual peer-reviewed publications and grey literature that focus on simulation in radiography education will be considered for inclusion. We will exclude publications with non-research study designs (e.g., editorial, discussion/opinion papers, guidelines, letters, and non-systematic reviews). All empirical and conceptual publications must have an abstract and aim clearly stated. No language or year restrictions will be applied, and we will not apply any restrictions regarding the status of publication. In line with the Joanna Briggs Institute Reviewer’s Manual [37], the detailed inclusion criteria of this scoping review are specified as the population, concept, context, and types of sources of evidence (Table 1).

**Table 1:** Study eligibility

	Inclusion criteria
**Population**	• Radiography students, both undergraduate and postgraduate • Faculty in radiography education • Radiography clinicians and clinical supervisors/clinical educators/instructors
**Concept**	• All types of simulation (e.g., integrated simulators, simulated patients, simulated environments, virtual reality and haptic systems, computer-based systems, part-task trainers) • All types of learning design/pedagogical methods • All types of learning outcomes (e.g., knowledge, skills, competence, generic skills, attitudes, self-efficacy) • All types of patient outcomes
**Context**	• Institutions educating radiographers (higher education institutions/universities, simulation labs or centers, hospitals) • Different professional radiography specializations: medical radiation sciences including the disciplines of radiography, digital/conventional radiography, interventional radiography, computed tomography, magnetic resonance imaging, radiation therapy, medical dosimetry, mammography, sonography/ultrasound, nuclear medicine
**Types of sources of evidence**	All empirical and theoretical/conceptual peer-reviewed publications and grey literature that focus on simulation in radiography education

### Search strategy and information sources

We will search MEDLINE, Embase, Epistemonikos, The Cochrane Library, ERIC and Scopus. To identify grey literature, we will search OpenGrey and Google Scholar. We will search the reference lists and citations of included studies to identify additional, relevant references. The searches will be re-run just before the final analyses to retrieve further studies for inclusion.

We developed a comprehensive search strategy for Ovid MEDLINE in collaboration with an experienced research librarian. An example of a full electronic search using search terms for simulation in radiography education in the Ovid MEDLINE yielded 1641 articles on January 9, 2020 (Additional file 2). We will adapt/use the search strategy used for the Ovid MEDLINE to each database. As the search strategy for scoping reviews is considered an iterative process [32–34], we will evaluate the initial search results and evaluate needs for improvement during the review process. Records will be exported to EndNote X9 [38] to enable data management, removal of duplicates, and retrieving full texts.

### Study selection

For the selection of eligible studies, we will use the Rayyan screening tool [39]. Based on the inclusion criteria (Table 1), two review authors will independently screen the titles and abstracts from the retrieved studies. Two review authors will then independently assess the acquired full-text publications for eligibility. Any disagreements regarding eligibility will be resolved by discussion among the two review authors, and a third reviewer will resolve disagreement if needed. If full-text articles are excluded, the reasons will be presented in an appendix. To ensure consistency and reliability in the study selection process, we will pilot the study selection using a random selection of 50 titles and abstracts from the literature search before we start formal screening. We will revise until we achieve a percentage agreement of > 80% across reviewers. The search decision process will be illustrated using a flow chart, as recommended in the PRISMA statement [40].

### Data charting process

Based on the population, concept, context, and types of sources of evidence as outlined in Table 1, the research team will develop a data extraction form using spreadsheets. Prior to the full data extraction, we will pilot the data extraction form using a sample of 10 studies to determine agreement within the research team, and as such, this will be an iterative process. Two review authors will then independently read and extract data from each included study using this data extraction form. In line with the purpose of this scoping review, we plan to extract the following data:

1. *Population*: study population (student, clinician, faculty), age, sex, level of education (year of study, undergraduate, postgraduate), inclusion and exclusion criteria, needs assessment (e.g., equipment, human resources), number of participants in intervention group/control group, sample size, and data about previous experience with simulation
2. *Concept*: type of intervention (scenario/task/activity, facilitative approach (pre- and debriefing/feedback), manikin or standard patient intervention, virtual reality), overall aim of the simulation (learning outcomes), type of skills, assessment after training (formative or summative evaluation), pedagogical rationales, integrated into curriculum (yes/no), duration (h), fidelity (equipment, environmental and psychological fidelity), settings (educational or healthcare institution or others), comparator, type of outcomes (educational, patient), cost measures used (yes/no), and type of cost measures
3. *Context*: type of institution performing the simulation and type of radiography specializations
4. *Types of sources of evidence*: title, year of publication, volume, author, country, study objective/purpose, type of study, research method (design, number of study participants/sample size, data collection), results, and conclusions

### Analysis of the evidence

In this review, we aim to present an overview and a narrative account of all studies included. We will present our results in two ways. Firstly, we will quantitatively summarize the data related to the extent, nature, and distribution of studies. This simple numerical analysis will provide an overview, and it will point to significant knowledge gaps. Secondly, we will use content analysis [41] to map the different simulation interventions and learning design elements reported (e.g., teaching and learning activities, curriculum, pedagogical theory, assessment strategies, and learning outcomes). Reporting guidelines for interventions (e.g., [42]) will be used to structure the presentation of the reported interventions. We will use Kirkpatrick’s four-level model [43] as a framework for the analysis of the different learning outcomes reported.

## Discussion

To the best of our knowledge, this will be the first scoping review identifying the research published on simulation in radiography education. This process will provide an overview of the current state of evidence in research about simulation in radiography education, and we will be able to identify in which research areas systematic reviews or primary research are needed.

The strength of this review is the use of a transparent and reproducible procedure. In our protocol, we have presented a detailed description of population, concept and context, data sources, search strategy, data extraction, and analysis*.* We will not limit the review to only certain kinds of simulation or settings because the radiography profession performs a wide range of clinical tasks, including image diagnostic and treatment procedures combined with patient-related care. We anticipate that this review will be useful for stakeholders within radiography education, both in the academic and clinical settings. Our search strategy is broad, which may result in a high number of redundant texts or publications. The search terms may be changed or expanded during search process due to the iterative method. In this scoping review, we will not assess the impact of simulation intervention, nor the quality of the identified interventions.

### Ethics and dissemination

Ethical approval or consent to participate is not required, as data in this study consists of published studies, and not individual data from human or animal participants. The research project will be carried out in accordance with the Helsinki Declaration.

### Funding

We will report sources of funding for the included sources of evidence in the studies. For the proposed scoping review, there is no declaration of funding.

## Supplementary Information

**Additional file 1.** PRISMA-P checklist

**Additional file 2.** Full electronic search in one database

## Data Availability

Not applicable as this is a protocol for a scoping review. Search strategy and preliminary search result will be available as an additional file.

## Abbreviations

WHO: World Health Organization
JBI: Joanna Briggs Institute
PRISMA-P: Preferred Reporting Items for Systematic Review and Meta-Analysis Protocols
PRISMA-ScR: Preferred Reporting Items for Systematic reviews and Meta-Analyses extension for Scoping Reviews
EFRS: European Federation of Radiographer Societies
EQF: European Qualifications Framework
