# Supplementary material for: Research on simulation in radiography education: a scoping review protocol
Source: Syst Rev. 2020 Nov 21;9:263. doi: 10.1186/s13643-020-01531-2 (PMC7680590; doi:10.1186/s13643-020-01531-2)
Supplement: Supplementary file 2 — Additional file 2. Full electronic search in one database [file 13643_2020_1531_MOESM2_ESM.docx]

**Additional file 2**: ***Full electronic search in one database***

Search terms for simulation in radiography education accomplished in *Ovid MEDLINE* on January 9, 2020 *are the following:* Search for: 18 and 21 and 22

Results: 1641

Database: Ovid MEDLINE(R) and Epub Ahead of Print, In-Process & Other Non-Indexed Citations and Daily <1946 to November 05, 2019> Search Strategy:

--------------------------------------------------------------------------------

1 Video Games/ (4987)

2 Internet/ (70386)

3 exp Educational Technology/ (107565)

4 exp Simulation Training/ (8057)

5 Computer Simulation/ (182948)

6 Virtual Reality/ (1280)

7 Computer-Assisted Instruction/ (11623)

8 User-Computer Interface/ (36240)

9 ((computer* or digital* or hybrid or blended or mixed mode or distan* or remote* or electronic or mobile or

online* or interactiv* or multimedia or internet or web* or virtual* or game* or gaming or videogame* or videogaming)

adj3 (classroom* or course* or educat* or instruct* or facilitat* or learn* or lecture* or simulat* or train* or teach* or tutor* or platform* or pedagog* or competenc*)).tw,kw. (76180)

10 ((educat* or instruct* or learn* or simulat* or train* or teach* or interactiv*) adj2 technolog*).tw,kw. (5343)

11 ((simulated or augmented or mediated) adj3 (realit* or world* or environment*)).tw,kw. (6019)

12 (virtual adj2 (realit* or system*)).tw,kw. (9307)

13 (patient* adj1 simulat*).tw,kw. (4076)

14 virtual patient*.tw,kw. (922)

15 standardi* patient*.tw,kw. (3152)

16 ((technical or non-technical) adj skill*).tw,kw. (4483)

17 (simulat* adj3 (course* or educat* or instruct* or learn* or train* or teach* or facilitat* or platform* or

high-fidelity or low-fidelity or interdisciplin* or interprofession* or manikin* or mannikin* or mannequin* or multidisciplin*)).tw,kw. (14350)

18 or/1-17 (460199)

19 Radiography/ (316213)

20 (radiograph* or radiolog* or radiation therap* or radiation technologist* or radiology technologist* or radiology

technician* or radiation technician* or imaging technologist* or diagnostic technologist* or nuclear technologist* or medical dosimetr* or computer tomograph* or magnetic resonance imaging or mammograph* or medical imaging or sonograph* or nuclear medicine or x-ray or scinitgraph*).tw,kw. (1066764)

21 19 or 20 (1271387)

22 (student* or graduate* or postgraduate* or undergraduate* or education or school*).tw,kw. (831805)

23 18 and 21 and 22 (1612)
